# Systemic contact dermatitis in a family: Case report

**DOI:** 10.1097/MD.0000000000039272

**Published:** 2024-08-16

**Authors:** Guang-mei Sun, Ting-ting Li, Jin-mei Zhang, Hai-yan Liu, Yan-li Xu, Bao-xiang Zhang

**Affiliations:** a Department of Dermatology, Yidu Central Hospital of Weifang, Qingzhou, Weifang, Shandong Province, China.

**Keywords:** benzalkonium chloride, systemic contact dermatitis

## Abstract

**Background:**

Systemic contact dermatitis (SCD) is an allergic inflammatory skin disease. We report that 3 family members developed SCD after exposing to laundry detergent containing benzalkonium chloride, which is rare. SCD caused by benzalkonium chloride has been reported. However, Similar symptoms in the whole family caused by it have not been reported yet. In our case, a 36-year-old man was diagnosed as SCD, and his symptoms had not controlled after 7 days treatment, until he stopped dressing the clothes washed by the laundry detergent containing benzalkonium chloride. It was interesting that both his wife and the daughter developed SCD successively, and they have not exposed to any haptens besides the benzalkonium chloride in the laundry detergent.

**Methods:**

Dermoscopic examination showed bright-red background, focal branching vessels and white scales. HE staining from the lesion revealed hyperkeratosis and parakeratosis, focal subcorneal microabscess, ocal hyperkeratosis, koilocyte in the epidermis, and erythrocyte extravasation, fibroplasia, hyaline degeneration and scattered aggregates of lymphocytes in the dermis. Then path test was performed 1 month after recovery with benzalkonium chloride 0.05% and 0.1% in petrolatum.

**Results:**

Stop the laundry detergent containing benzalkonium chloride. The symptoms had controlled after they stopped the laundry detergent containing benzalkonium chloride.

**Conclusion:**

The case highlights that benzalkonium chloride with very low concentration and repeated exposure may be an active agent of SCD. It is of the utmost importance to pay close attention to patients presenting with similar symptoms within the family. A thorough examination of the medical history is essential to determine the underlying cause.

## 1. Introduction

Systemic contact dermatitis (SCD) is an allergic inflammatory skin disease, which clinical manifestation are various such as erythema, vesicular hand eczema, maculopapular eruption, erythema multiforme, vasculitis, flexural dermatitis or the baboon syndrome.^[1]^ SCD may occur in persons when they are exposed to the haptens including metals, medications, aromatic substances and foods, by transcutaneous, inhalational, oral, transrectal, intravenous, intramuscular route and so on.^[2]^

## 2. Case report

**Figure 1.**
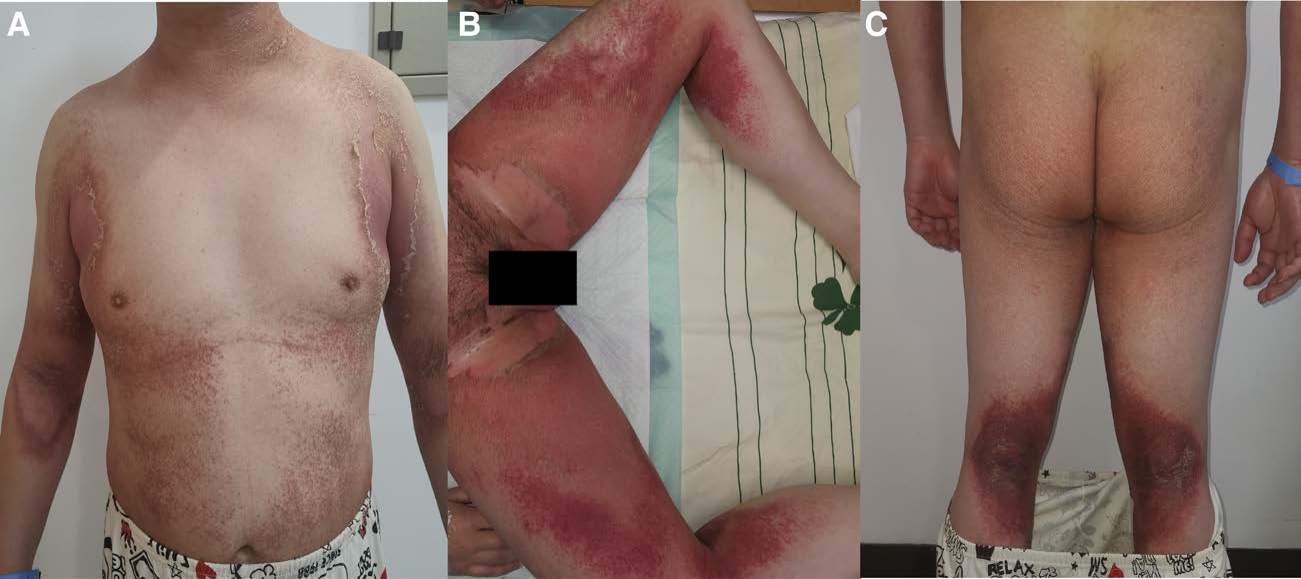
The eruption of the man in trunk (A), thigh (B), and popliteal fossa (C).

**Figure 2.**
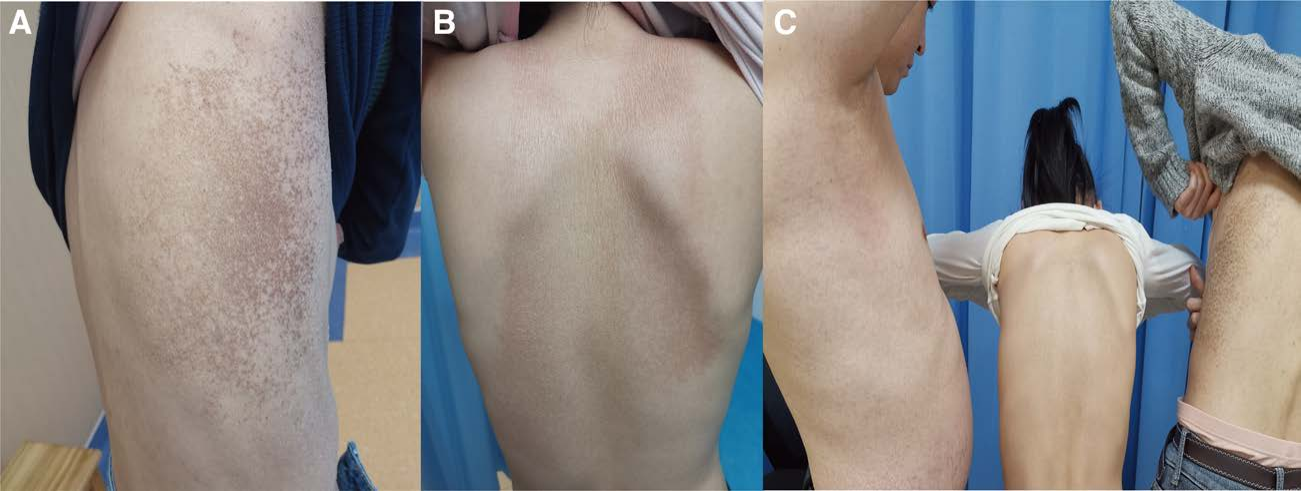
The eruption of the wife in trunk (A), The eruption of the daughter in dorsum (B), the families come back to our department 3 weeks later after the first examination of the husband (C).

A 36-year-old man presented to our department because of an acute symmetry eruption of the neck, trunk, axillary, thigh, genitals, and popliteal fossa consisting of erythematous plaques with pustules and slightly scaring (Fig. 1). And the patient had previous erythematous plaques on his genitals 1 month ago which fade quickly after a few days of treatment. The levels of normal routine blood tests, total serum IgE levels, liver function tests, antinuclear antibodies and C reactive protein of this patient were normal. He was treated with intravenous methylprednisolone 80 mg/d, cimetidine and vitamin C, followed by oral antihistamines and topical corticosteroids. It’s worth noting that, 6 days later his wife visited to our department complained similar eruption on her cubital fossa, popliteal fossa, trunk and thigh (Fig. 2A). Meanwhile the eruption of the husband sustained developed. They both have not exposed to any plants, metals, nickel, cobalt, mercury, chromate, gold etc. However, they use a new laundry sanitizer containing benzalkonium chloride 2.5 ± 0.25% (W/W) in recent 5 months to avoid the influence of COVID-19. So the laundry sanitizer and all the clothing washed by the sanitizer were stopped from the seventh day, and then the condition of the parents improved gradually. Furthermore, 2 weeks later the daughter came home from school and visited to our department for the slightly erythematous and scaling on her chest and back for 5 days (Fig. 2B), particularly in the last 2 weeks the daughter lived in school all the time, and her cloths were washed by the laundry sanitizer. Then the families come back to our department together 3 weeks later (Fig. 2C), and within the 3 months of follow-up without the sanitizer, there was no recurrence of the 3 family members. In summary, all patients showed significant improvement in symptoms after discontinuing the use of the detergent containing benzalkonium chloride.

### 2.1. Patch test, dermoscopic examination and HE staining

**Figure 3.**
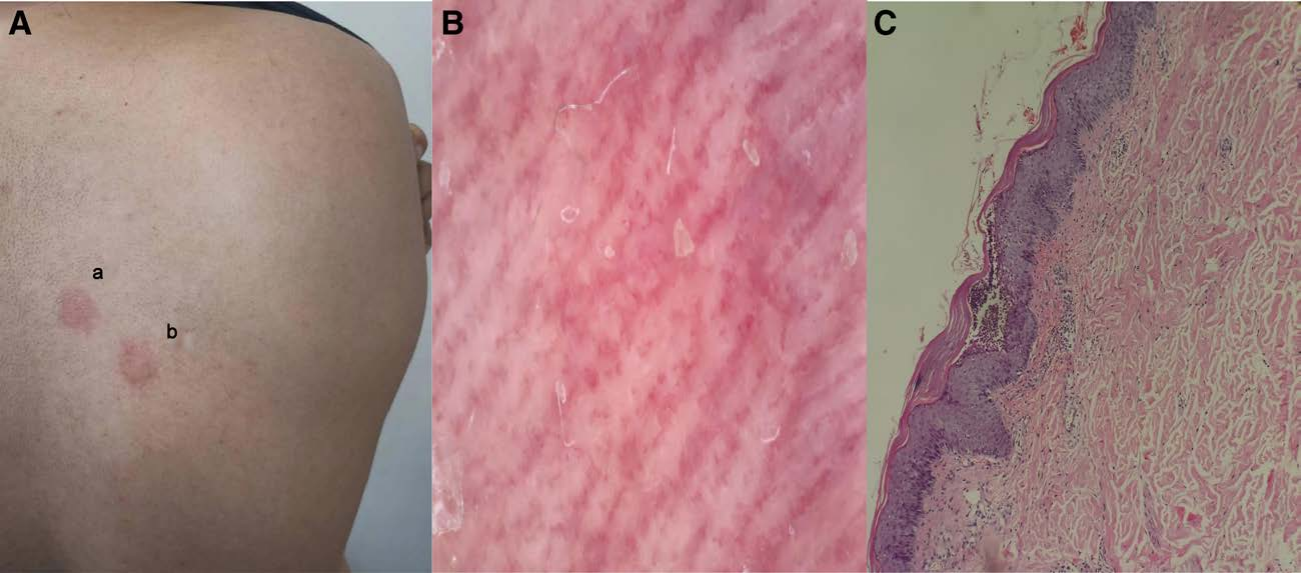
The husband performed path testing with benzalkonium chloride 0.05% (a) and 0.1% (b) in petrolatum (A). The dermoscopic (B) and pathological (HE × 40) (C) feature of the lesion.

Then patch test was performed 1 month after recovery with benzalkonium chloride 0.05% and 0.1% in petrolatum (Fig. 3A). Dermoscopic examination showed bright-red background, focal branching vessels and white scales. HE staining from the lesion revealed hyperkeratosis and parakeratosis, focal subcorneal microabscess, ocal hyperkeratosis, koilocyte in the epidermis, and erythrocyte extravasation, fibroplasia, hyaline degeneration and scattered aggregates of lymphocytes in the dermis (Fig 3B, C).

## 3. Discussion

SCD may occur in contact-sensitized persons when they are reexposed to the same haptens systematically. The precise mechanism underlying SCD reactions remains yet to be clearly defined.^[1]^ There are several studies supporting a T-cell mediated type 4 hypersensitivity reaction and CD8 + CD45RO + CLA + T cells involved in the pathogenesis of SCD.

Benzalkonium chloride, a quaternary ammonium cationic surfactant, is a well-recognized irritant and an uncommon cause of allergic contact dermatitis. And positive test reactions were observed significantly more often in the disinfectants series compared with the topical drugs series and ophthalmics series, indicating that benzalkonium chloride is also a contact allergen, albeit a very rare one.^[3]^ Researchers have conducted a retrospective study in Australia. Of the 7390 patients with suspected contact dermatitis who were patch tested to benzalkonium chloride, 26 patients had a clinically relevant positive reaction (0.4% of total patch tested). And the common sources of exposure were ophthalmic drops, topical antiseptic preparations, cosmetics, disinfectant solutions, hand sanitizers, and hand washes.^[4]^ Another report demonstrated a child developed erythema multiforme-like reaction after using potty washed by benzalkonium chloride.^[5]^ Some researchers suggest sensitization to benzalkonium chloride is more likely occurred when used as disinfectant with a concentration >1%.^[3]^

In our case, the families have not exposed to the haptens besides the laundry sanitizer containing benzalkonium chloride and have the similar symptoms successively. The symptoms of the husband had not controlled after 7 days treatment, until he stopped the laundry detergent containing benzalkonium chloride. So we supposed benzalkonium chloride as the cause of the case. According to the operation guide, the clothes were washed by the benzalkonium chloride with a concentration from 0.03% to 0.06% and it’s worth noting that the clothes were rinsed in washing machine. So it suggests that SCD may also be induced by benzalkonium chloride with long-term exposure at very low concentrations.

## 4. Limitations

This study presents a descriptive account of the cases in question and does not delve into verifying the underlying mechanisms. Further research is required to understand these mechanisms better. The case report focuses on a family of 3, resulting in a relatively small sample size. This limits the generalizability of the findings. We will continue to collect additional cases and further investigate the mechanism of benzalkonium chloride-induced SCD. Future studies will aim to provide a more comprehensive understanding of this condition.

## 5. Conclusion

It is interesting that the 3 family members developed SCD after exposing to laundry detergent containing benzalkonium chloride. And the case highlights that benzalkonium chloride with very low concentration and repeated exposure may be an active agent of SCD, and the underling mechanism needs further study.

## Author contributions

**Conceptualization:** Guang-mei Sun, Hai-yan Liu.

**Data curation:** Guang-mei Sun, Hai-yan Liu, Yan-li Xu.

**Formal analysis:** Ting-ting Li, Yan-li Xu.

**Investigation:** Jin-mei Zhang.

**Project administration:** Ting-ting Li, Jin-mei Zhang.

**Resources:** Ting-ting Li, Bao-xiang Zhang.

**Software:** Jin-mei Zhang.

**Supervision:** Yan-li Xu, Bao-xiang Zhang.

**Validation:** Bao-xiang Zhang.

**Writing – original draft:** Guang-mei Sun, Ting-ting Li, Hai-yan Liu, Yan-li Xu, Bao-xiang Zhang.

**Writing – review & editing:** Bao-xiang Zhang.
